# User-friendly and ultra-stable all-inclusive gold tablets for cysteamine detection†

**DOI:** 10.1039/d3ra03073c

**Published:** 2023-06-29

**Authors:** Muna Al-Kassawneh, Zubi Sadiq, Sana Jahanshahi-Anbuhi

**Affiliations:** a Department of Chemical and Materials Engineering, Gina Cody School of Engineering, Concordia University Montréal Québec Canada sana.anbuhi@concordia.ca

## Abstract

To date, a range of nanozymes has been reported for their enzyme-mimicking catalytic activity such as solution-based sensors. However, in remote areas, the need for portable, cost-effective, and one-pot prepared sensors is obvious. In this study, we report the development of a highly stable and sensitive gold tablet-based sensor for cysteamine quantification in human serum samples. The sensor is produced in two steps: synthesis of a pullulan-stabilized gold nanoparticle solution (pAuNP-Solution) using a pullulan polymer as a reducing, stabilizing, and encapsulating agent and then, casting the pAuNP-Solution into a pullulan gold nanoparticle tablet (pAuNP-Tablet) by a pipetting method. The tablet was characterized by UV-vis, DLS, FTIR, TEM, and AFM analyses. The pAuNP-tablet exhibited a high peroxidase-mimetic activity *via* a TMB–H_2_O_2_ system. The presence of cysteamine in the system introduced two types of inhibition which were dependent on the cysteamine concentration. By determining Michaelis–Menten's kinetic parameters, we gained mechanistic insights into the catalytic inhibition process. Based on the catalytic inhibition capability of cysteamine, the limit of detection (LoD) was calculated to be 69.04 and 82.9 μM in buffer and human serum samples, respectively. Finally, real human serum samples were tested, demonstrating the applicability of the pAuNP-Tablet for real-world applications. The % R values in human serum samples were in the range of 91–105% with % RSD less than 2% for all replicas. The stability tests over 16 months revealed the ultra-stable properties of the pAuNP-Tablet. Overall, with a simple fabrication method and a novel employed technique, this study contributes to the advancement of tablet-based sensors and helps in cysteamine detection in clinical settings.

## Abstract 2

To date, a range of nanozymes has been reported for their enzyme-mimicking catalytic activity such as solution-based sensors.

## Graphical abstract

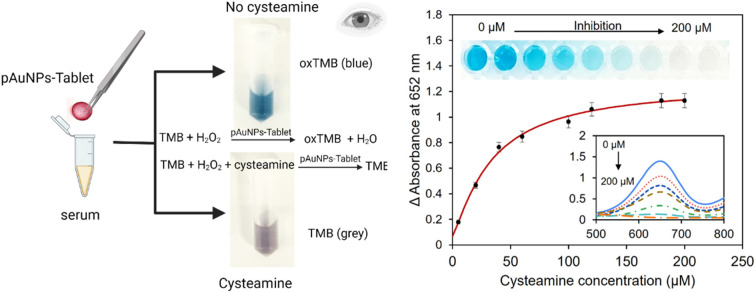

## 1. Introduction

Cysteamine, a type of amino acid medication that is used to treat cystinosis, works by interacting with crystalline cysteine to reduce the buildup of intraliposomal cystine.^1^ According to some reports, one case per 100 000/200 000 live births suffers from cystinosis.^2^ In living organisms, the neuronal structure and/or function can be maintained due to cysteamine's neuroprotective and antioxidation properties to treat neurodegenerative diseases.^3,4^ The concentration of cysteamine has been documented to be in the low micromolar range in organs such as the liver, kidney, and brain.^5^ Elevated levels can cause side effects such as Ehlers–Danlos syndrome, high alkaline phosphatase, ulcers/bleeding in the gastrointestinal tract, and idiopathic intracranial hypertension (IIH), which can cause ringing in the ears and loss of vision along with dizziness and nausea.^6^ Therefore, the daily intake of cysteamine for patients with cystinosis based on body weight is (1.3–1.95)g/m^2^/day distributed over 4–2 doses.^7,8^ However, serum/plasma cysteamine levels in healthy cystinosis patients should be monitored to be ∼150 μM after 30–60 minutes of cysteamine digestion.^9^ Due to the adverse effects of cysteamine in clinical settings, it is critical to develop an economic, transportable, and easy-to-operate bioanalytical sensor for cysteamine detection in serum samples.

Nowadays, cysteamine is used to treat a wide range of neurodegenerative diseases such as Parkinson's,^10^ Huntington's,^11^ and Alzhamier's.^12^ These diseases can be detected rapidly, precisely, and economically using point-of-care (POC) analytical devices. Numerous appealing detection systems such as microfluidic lateral flow paper-based assays^13^ and smart sensing devices^14^ have been launched. These platforms, however, present several challenges yet. For example, microfluidic lateral flow paper-based assays have issues with sample leakage,^15^ and are less sensitive,^16^ at the same time, intelligent detection devices have complicated assemblies and sophisticated data analysis, which make them costly and less user-friendly.^17^ In recent studies, the incorporation of reagent encapsulation techniques has been preferred to ensure swift detection in a liquid phase by discharging enclosed reagents during the testing process.^18–21^ This combined method not only yields a sensitive detection system but also merges the beneficial aspects of point-of-care analytical devices. Therefore, scientists and researchers are keen to investigate novel platforms for point-of-care devices.

Recently, significant focus has been placed on colorimetric techniques that rely on the localized surface plasmon resonance (LSPR) feature of gold nanoparticles.^22^ The ease of use and relatively quick signal reading make colorimetric methods appealing. The interaction between analytes and nanoparticles affects the color difference between dispersed and aggregated nanoparticles, which is fundamental to colorimetric detection.^23^ Nonetheless, the accurate control of nanoparticle size and inter-particle distance, which are challenging variables in LSPR-based colorimetric techniques,^24^ is necessary to generate colorimetric signals for analyte detection. Additionally, the stability of nanoparticles linked to aggregation is another limitation of colorimetry based on the LSPR of gold nanoparticles.^25^ Consequently, catalysis-based colorimetric techniques for identifying various analytes are gaining interest as they overcome these limitations.^26^ This is because, the catalytic performance of gold nanoparticles is usually monitored calorimetrically *via* the redox properties of gold, which allow it to act as an electron acceptor and donor, facilitating the transfer of electrons between H_2_O_2_ and the substrate. The generation of a single type of product *via* this reduction reaction simplifies the monitoring of the reaction's progress through UV-visible spectrophotometric measurements.^27^

Nanozymes are inorganic nanomaterials that are credited to mimic the catalytic activity of natural enzymes. Till date, various nanozymes including metals, metal-oxides, metal sulfides, and carbon-based materials have been documented for their catalytic activity that mimics enzymes.^28^ A recent assessment revealed that more than 200 research facilities worldwide are engaged in enhancing the development of novel nanozymes and broadening their scope of use.^29^ Among the various uses and basic research, a particular aspect that is receiving quick recognition is the capacity to regulate the catalytic function of nanozymes to either improve or block and temporarily or permanently hinder their catalytic activity. To this end, metal ions^30,31^ and biomolecules such as ATP,^32^ cysteine,^33^ and glutathione^34^ have all been effectively devoted to modulating the catalytic activity of nanozymes. As observed, ATP enhanced the catalytic efficiency of nanozymes,^35^ whereas cysteine, an amino acid, hindered their catalytic performance.^36^ These inhibitory molecules attach either to the enzyme's active site or near it, leading to a reduction (temporary) or total loss (permanent) in catalytic effectiveness.^37^ When it comes to temporary inhibition, the inhibitor molecule attaches reversibly to the enzyme causing a temporary reduction in the catalytic rate. In contrast, irreversible inhibitors bind to the enzyme and obstruct its activity on a permanent basis.^38^ Moreover, reversible inhibition has provided a profound understanding of the reversible inhibition mechanism (competitive, uncompetitive, or non-competitive) by computing the steady-state kinetics parameters, namely Michaelis–Menten's constant (*K*_m_) and maximum reaction velocity (*V*_max_).^39^

In the current study, we used the developed pAuNP-Tablet as a nanozyme sensor to evaluate their molecular interaction with cysteamine. To accomplish this, we developed a simple solution-based path to prepare a pAuNP-Solution. Then, the premeasured amount of pAuNP-Solution was cast into tablets by a pipetting method. The pAuNP-Tablet displays a catalytic behavior higher than that of natural enzymes, with the colloidal gold phase exhibiting superior peroxidase-mimicking catalytic activity, while the pullulan's unique capability to encapsulate gold provides the tablet platform. The pAuNP-Solution and pAuNP-Tablet were characterized by UV-vis spectroscopy, dynamic light scattering (DLS), transmission electron microscopy (TEM), Fourier transform infrared (FTIR) spectroscopy, and atomic force microscopy (AFM) analyses. Introducing the pAuNP-Tablet to cysteamine caused temporary/permanent inhibition of the catalytic activity, where the degree of inhibition was dependent on the cysteamine concentration. The kinetics parameters of the Michaelis–Menten equation (*V*_max_ and *K*_m_) were further examined to establish the type of inhibition that occurred in the presence of different concentrations of cysteamine. Additionally, the inhibition of another catalytic activity (4-nitrophenol to 4-aminophenol conversion) was investigated in the presence and absence of cysteamine. Based on the strong specificity of interactions between cysteamine and pAuNPs, we developed a simple sensor that detects cysteamine levels in the presence of other potential interferences. To the best of our knowledge, this is the first time that cysteamine detection was accomplished by applying the inhibition of the peroxidase-like activity of a tablet-based sensor made of pullulan-capped gold nanoparticles. Lastly, we demonstrate the applicability of the peroxidase tablet sensor in the detection of cysteamine in complex matrices in artificial and real human serum samples.

## 2. Materials and methods

### 2.1 Chemicals

All reagents and chemicals were of analytical grade and used as received. Gold(iii) chloride solution (30 wt% in dilute HCl), cysteamine (C_2_H_7_NS), 3,3′,5,5′-tetramethylbenzidine (TMB), dimethyl sulfoxide (DMSO)(C_2_H_6_OS), sulfuric acid (H_2_SO_4_), trisodium citrate (C_6_H_5_Na_3_O_7_), sodium phosphate monobasic monohydrate (NaH_2_PO_4_·H_2_O), sodium phosphate dibasic (Na_2_HPO_4_), hydrogen peroxide (H_2_O_2_), citric acid (C_6_H_8_O_7_), sodium hydroxide (NaOH), sodium borohydride (NaBH_4_), 4-nitrophenol (C_6_H_5_NO_3_), methionine, asparagine, glycine, glutathione, aspartic acid, cysteine, and arginine were purchased from Sigma-Aldrich, USA. Pullulan (average *M*_w_: ∼200 kDa) was obtained from Polyscience, Inc., USA. Artificial serum (99.99% purified) was purchased from Biochemzone, USA. Real human serum samples were attained from a clinical lab in Montreal, CA. Deionized water obtained from Sigma Aldrich, USA was used to prepare all solutions. A carbon steel tray (Betty Crocker) was obtained from a local store, in Canada.

### 2.2 Synthesis and fabrication of the pAuNP-Solution and pAuNP-Tablet

**Scheme 1:**
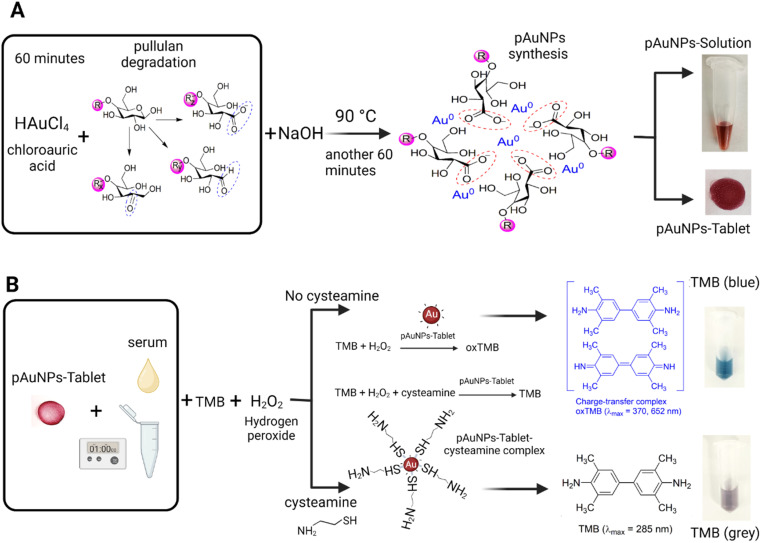
Representation of the synthesis of the pAuNP-Solution/pAuNP-Tablet and the mechanism of cysteamine detection. (A) Production of tablets containing gold nanoparticles stabilized with pullulan (pAuNP-Tablet) was achieved by using a pAuNP-Solution. To start, pullulan was subjected to NaOH-mediated degradation at a temperature of 70 °C for 60 minutes. The next step involved the addition of a solution of HAuCl_4_ and the heating of the reaction mixture to 90 °C for further 60 minutes to synthesize the pAuNP-Solution. Finally, the pAuNP-Solution was pipetted onto a non-stick tray to form the pAuNP-Tablet. The tablets were then dried in an oven at 50 °C for 30 minutes or left to dry at room temperature for 24 hours. (B) Mechanism of peroxidase-like activity inhibition in the presence of cysteamine. In the absence of cysteamine, the active sites of pAuNPs surface are free, so that TMB can attach easily to the pAuNP surface. Consequently, the charge transfer from the surface of pAuNPs to H_2_O_2_ generates the radical ˙OH, resulting in the formation of oxidized TMB (oxTMB) (blue color). Upon incubating pAuNPs with cysteamine, a pAuNP–cysteamine complex is formed, which blocked the active sites on the surface of pAuNPs. When TMB is added because the surface is blocked with cysteamine, no charge transfer can leave the surface of pAuNPs causing the inhibition of the formation of oxTMB. Hence, the colorless-grey color will not develop into a blue color.

The glassware was cleansed using a freshly prepared solution of *aqua regia* and rinsed three times with deionized water. Monodisperse Au nanoparticles were generated by using a pullulan polymer and following the protocol described in the literature, with some modifications.^40^ First, the treated pullulan was produced by mixing 3% (w/v) pullulan (30 mL) and 1% (w/v) NaOH (12.5 mL). The solution was warmed up to 70 °C for 60 minutes. Then, a 2.5 mM HAuCl_4_ solution (7.5 mL) was added, and the reaction mixture was agitated at 90 °C for further 60 minutes. The concentration of the pullulan-capped AuNP solution (pAuNP-Solution) was estimated to be ∼11 nM using Beer's law, which had an extinction coefficient (*ε*) of 1.876 × 10^8^ M^−1^ cm^−1^. The extension coefficient was calculated using the formula (ln *ε* = 3.32 ln *d* + 10.8).^41^ The absorbance was recorded at 520 nm using a UV-vis spectrophotometer (BioTek, Cytation 5, imaging reader). From the colloidal solution of pAuNPs, tablets (pAuNP-Tablet) were made. Scheme 1A represents the synthesis procedure of the pAuNP-Solution and pAuNP-Tablet. The weight of the pAuNP-Tablet was measured to be ∼1.2 mg using a digital scale (Sartorius brand).

### 2.3 Sensing of cysteamine in a phosphate buffer

At first, the peroxidase activity of the pAuNP-Tablet was evaluated by investigating its ability to oxidize a TMB substrate to a blue-colored product using H_2_O_2_ as an oxidizing agent. The detection system involved incubation of 300 μL of 50 mM Mcilvaine buffer, which is a citrate-phosphate buffer (pH 4), 1 pAuNP-Tablet (100 μL, 1.2 mg), 300 μL of 10 mM TMB, 100 μL of 1 mM H_2_O_2_ 25 °C for 20 minutes followed by UV-vis spectroscopic measurements using a 96-well plate reader (Ulti Dent Scientific, Canada) as a function of time. The pH of the Mcilvaine buffer solution was adjusted between 2 and 6 with citric acid or sodium phosphate dibasic using a pH meter (AB200 pH/mV/conductivity made from Fisher Scientific Accumet, Singapore). Reaction conditions such as pAuNP-Tablet nanozyme concentration, buffer pH, reaction temperature, TMB concentration, the colloidal ratio of pAuNPs to cast the tablets, and the cysteamine ratio to cause inhibition were optimized. This experiment was followed by the detection of cysteamine concentrations (0–200 μM) in the phosphate buffer (PB) (pH 7). In a typical procedure, a stock solution of cysteamine with various concentrations (0–200 μM) was prepared in PB at pH 7. Then, 1 pAuNP-Tablet and 100 μL cysteamine were incubated for 1 min and mixed using a vortex (Model# 945FIALUS, 50/60 Hz Fisherbrand, USA) followed by the addition of 300 μL 10 mM TMB, 300 μL 50 mM Mcilvaine buffer, and 100 μL 1 mM H_2_O_2_. The absorbance was collected at 652 nm after 20 minutes to measure the color intensity of the solution. To determine the reaction kinetics, different concentrations of cysteamine (0, 60, and 200 μM) were used with 1 pAuNP-Tablet. The zero-order rate constants were determined by plotting the maximal absorbance at 652 nm against reaction time (0–30 min). The reaction velocity was calculated using the Beer–Lambert law, where the absorbance value of oxidized TMB (oxTMB) was converted into product concentration using the molar extinction coefficient of oxTMB (39 000 M^−1^ cm^−1^). The Michaelis–Menten constant (*K*_m_) and initial maximum velocity of the reaction (*V*_max_) were calculated using non-linear curve fitting software (CurveExpert Professional, USA). The catalytic activity test was performed *via* the conversion of 4-nitrophenol (4-NP) (yellow) to 4-aminophenol (4-AP) (colorless) in the presence of pAuNP-Tablet with/without cysteamine. The reagents were prepared according to the literature procedure with some modifications.^42^ In separate test tubes, 1 mL of 0.1 mM of 4-NP and 0.5 mL of 0.1 M of NaBH_4_ were dissolved in deionized distilled water. Next, the pAuNP-Tablet and 100 μL of 0, 60, and 200 μM cysteamine were incubated and added. UV-vis spectroscopy was performed to measure the absorbance values and the kinetics of ratios was monitored based on the color change of the solution at 400 nm (yellow) and 300 nm (colorless). The apparent reaction constant (*k*_app_) was calculated using the formula reported by Azzam *et al.*:^43^ ln(*A*_*t*_/*A*_0_) = −*k*_app_*t*.

### 2.4 Sensing of cysteamine in artificial serum

The catalytic activity of the pAuNP-Tablet, which mimics peroxidase, was assessed in an artificial serum solution (pH 6.8) with different cysteamine concentrations (0–200 μM), as outlined in Section 2.3. The limit of detection (LoD) was determined using the formula 3*σ*/*μ*, where *σ* and *μ* represent the relative standard deviation of absorbance measurements and the slope of the linear calibration plot, respectively.

### 2.5 Interference study for the colorimetric detection of cysteamine

The effect of potential interferences such as methionine, asparagine, glycine, glutathione, aspartic acid, cysteine, and arginine was studied for our proposed cysteamine assay following the procedure mentioned in Section 2.3. All the interferants were used at 100 μM concentration in this experiment.

### 2.6 Analysis of cysteamine in human serum

To demonstrate the practical application of the pAuNP-Tablet sensor under real-life conditions, the detection of cysteamine was also conducted in real human serum samples taken from cystinosis patients (pH 6.5–7.0). Furthermore, a spiking test was conducted in actual human serum, and the method was validated by calculating the percent recovery (% R) using the formula reported by Sasikumar *et al.*:^44^ Rate of recovery (%) = (amount of cysteamine found (μM))/(amount of cysteamine added (μM)) × 100%.

## 3. Results and discussion

### 3.1 Characterization of pAuNPs

**Fig. 1:**
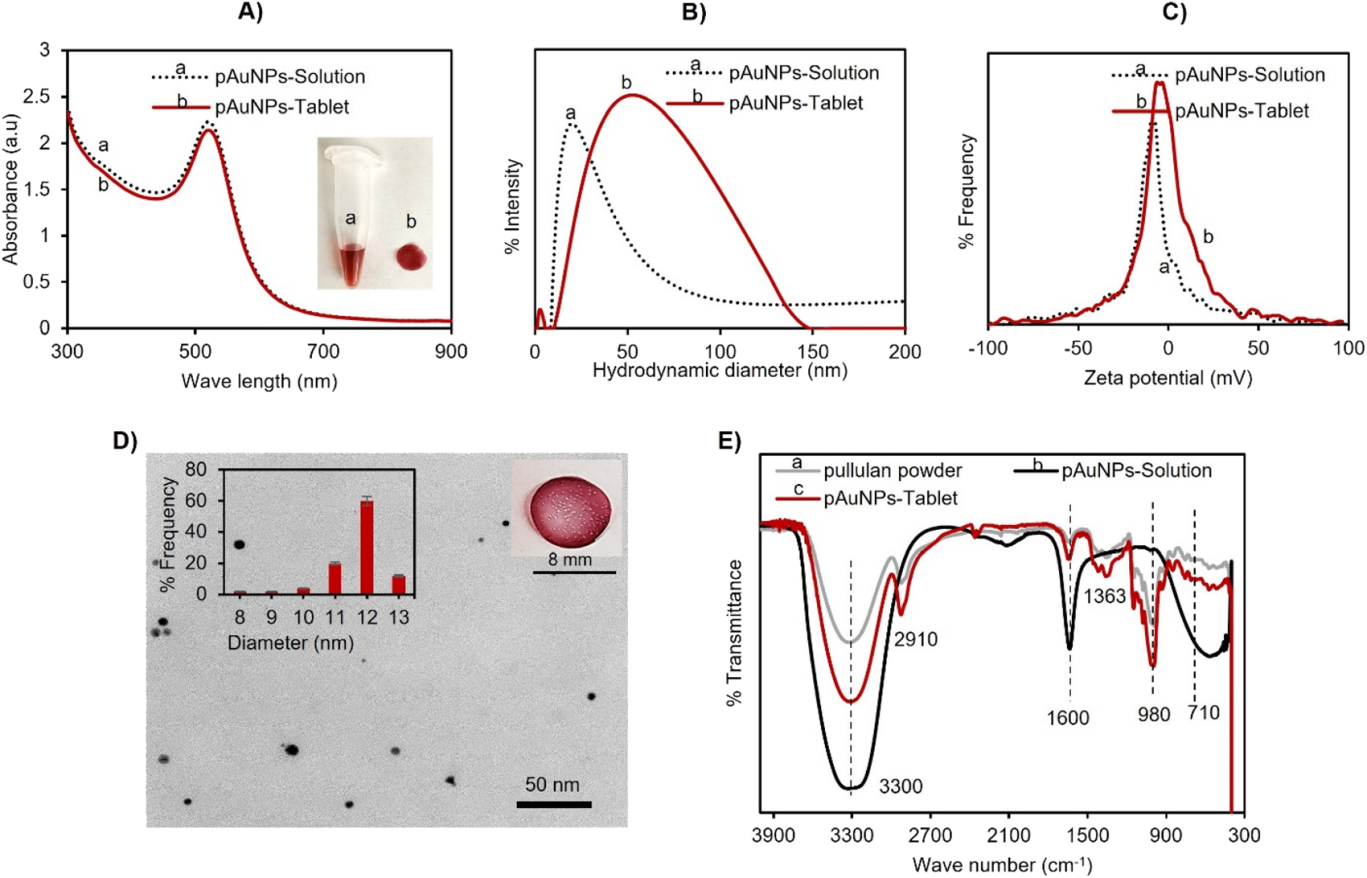
Characterization of the pAuNP-Solution and pAuNP-Tablet. (A) UV-vis spectroscopy showing a peak at 520 nm. (B) DLS analysis of the pAuNP-Solution and pAuNP-Tablet displays a little increase in the hydrodynamic diameter of the pAuNP-Tablet. (C) *ζ*-Potential analysis exhibits a little decrease in the *ζ*-potential value from −9 to −6. (D) TEM image analyses indicating spherical AuNPs with an average particle size of 12 ± 1.45 nm. (E) FTIR analyses showing that the pullulan polymer was well structured in the formation of the pAuNP-Tablet.

The pAuNP-Tablet sensor was established to identify cysteamine in human serum through the catalytic characteristics of the pAuNP-Tablet. The pAuNP-Solution was synthesized by mixing alkali-treated pullulan and a gold salt in water without any other reducing or capping agents. The degraded pullulan served as an effective reducing agent for tetrahydroauric chloride to synthesize AuNPs encircled by pullulan having carboxyl groups –C(=O) OH upon oxidation. The negatively charged carboxylate ions –COO^−^ properly encircled the AuNPs and thus functioned as a capping ligand. Furthermore, aside from being a reducing and stabilizing agent, the long polymeric chain of pullulan served as a reagent for tablet formation because of the film-forming quality of pullulan upon drying. The absorption spectra at 520 nm of the pAuNP-Solution and that of the pAuNP-Tablet presented the same particle size, as shown in Fig. 1A.

Next, the solid tablets were collected and characterized by DLS, TEM, and FTIR analysis. In DLS analysis, the hydrodynamic diameter of the pAuNP-Tablet and pAuNP-Solution was found to be 36.9 nm with 22% PDI (polydispersity index) and 91.77 nm with 24% PDI, respectively, as presented in Fig. 1B. Then, the zeta potential (*ζ*-potential) of the pAuNP-Tablet solution was compared with the pAuNP-Solution to see the particle's surface charge. The obtained values were −9.7 and −6 mV for the pAuNP-Solution and pAuNP-Tablet (Fig. 1C). The nanoparticle size and shape in the pAuNP-Tablet were confirmed by the TEM image, as shown in Fig. 1D. The image illustrates spherical shape particles having an average particle size of 12 ± 1.45 nm, which was also confirmed by the UV-vis absorption peak at 520 nm. The FTIR spectrum of pAuNP-Tablet was contrasted with pristine pullulan and alkali-degraded pullulan, which indicated the successful incorporation of pullulan chains around AuNPs, as shown in Fig. 1E. The FTIR analyses showed a broad absorption band at 3300 cm^−1^ due to O–H stretching, whereas other common absorption peaks for the pAuNP-Tablet and pure pullulan were at 2910 and 2917 cm^−1^, which were related to sp^3^ hybridization of C–H stretching. The absorption band at 1600 cm^−1^ was due to the O–H bending vibration of H_2_O molecules in alkali-treated pullulan. The absorption at 1363 cm^−1^ was related to C–O–H bending, while the peaks at 710 and 980 cm^−1^ revealed the presence of α-(1,4)-d-glucosidic and α-(1,6)-d-glucosidic bonds in the pAuNP-Tablet.^45^ Furthermore, the pAuNP-Tablet displays new absorption bands at 1350 and 1593 cm^−1^ due to the symmetrical and asymmetrical stretching vibrations of –COO^−^.^46^ Finally, to examine the pAuNP-Tablet surface morphology and its surroundings in the solid state, high-resolution surface images of the pAuNP-Tablet were acquired by AFM scanning probe microscopy. The pullulan matrix contained dispersed AuNPs, resulting in a red-colored tablet, as depicted in Fig. S1A.† The phase trace image in the figure revealed that the mean height of the AuNPs was between 10 and 30 nm, indicating that they were uniformly distributed and well preserved throughout the exopolysaccharide substance. The largest gold particle in the image had a diameter of 89.40 nm. For the surface roughness and texture description of the pAuNP-Tablet in 2D and 3D height profiles, refer to Fig. S1B–D.†

### 3.2 Mechanism of cysteamine detection using the pAuNP-Tablet

It is well known that the catalytic activity of gold nanoparticles is primarily influenced by their surface charge characteristics.^47^ During heterogeneous catalysis, the chemical bonds within the reacting molecules that are adsorbed on the active solid surface are broken and reformed, leading to the eventual release of products back into the liquid or gas phase. As a result, the presence of negatively charged carboxylate groups occupying the gold surface using the pullulan polymer as a labeling/capping agent can have an impact on the catalytic activity of gold nanoparticles. Thus, TMB was chosen in the assay because they have opposite charge characteristics.^48^ As a result, pullulan-capped gold nanoparticles, which are negatively charged, tend to attract positively charged amino groups of TMB electrostatically. These anionic pAuNPs will exhibit strong affinity to the TMB as a reaction substrate, thereby facilitating the charge transfer between pAuNPs and TMB.

The colorimetric test using tablets examined the enzyme-like activity of the negatively charged pullulan gold nanoparticle tablet, which mimics peroxidase, by utilizing H_2_O_2_ as an oxidant and TMB as a substrate. Peroxidase is an enzyme that decomposes H_2_O_2_. Thus, the pAuNP-Tablet was utilized to decompose H_2_O_2_ and produce hydroxyl radicals (˙OH), which are reactive oxygen species (ROS), in the detection system. The acidic conditions required for the TMB–H_2_O_2_ system were maintained using a Mcilvaine buffer. The ˙OH produced was unstable due to the presence of an unpaired electron, which was stabilized by interacting with the conduction band electrons of the pAuNP-Tablet. These interactions resulted in the peroxidase-like catalytic activity of the pAuNP-Tablet, leading to the oxidation of TMB and the formation of a blue-colored product. However, in the presence of cysteamine, the –SH group of cysteamine binds to the surface of pAuNPs *via* electrostatic attraction,^49^ resulting in the formation of a pAuNP-Tablet–cysteamine complex. As such, the aggregation of the pAuNP-Tablet in the presence of cysteamine was further examined. It was observed that the presence of cysteamine concentrations (0–400 μM) did not cause any aggregation of the pAuNP-Tablet until the concentration reached 500 μM, as displayed in Fig. S2.† Therefore, a hypothesis of the complex formation rather than an aggregation was suggested to inhibit the catalytic activity of the pAuNP-Tablet.

**Fig. 2:**
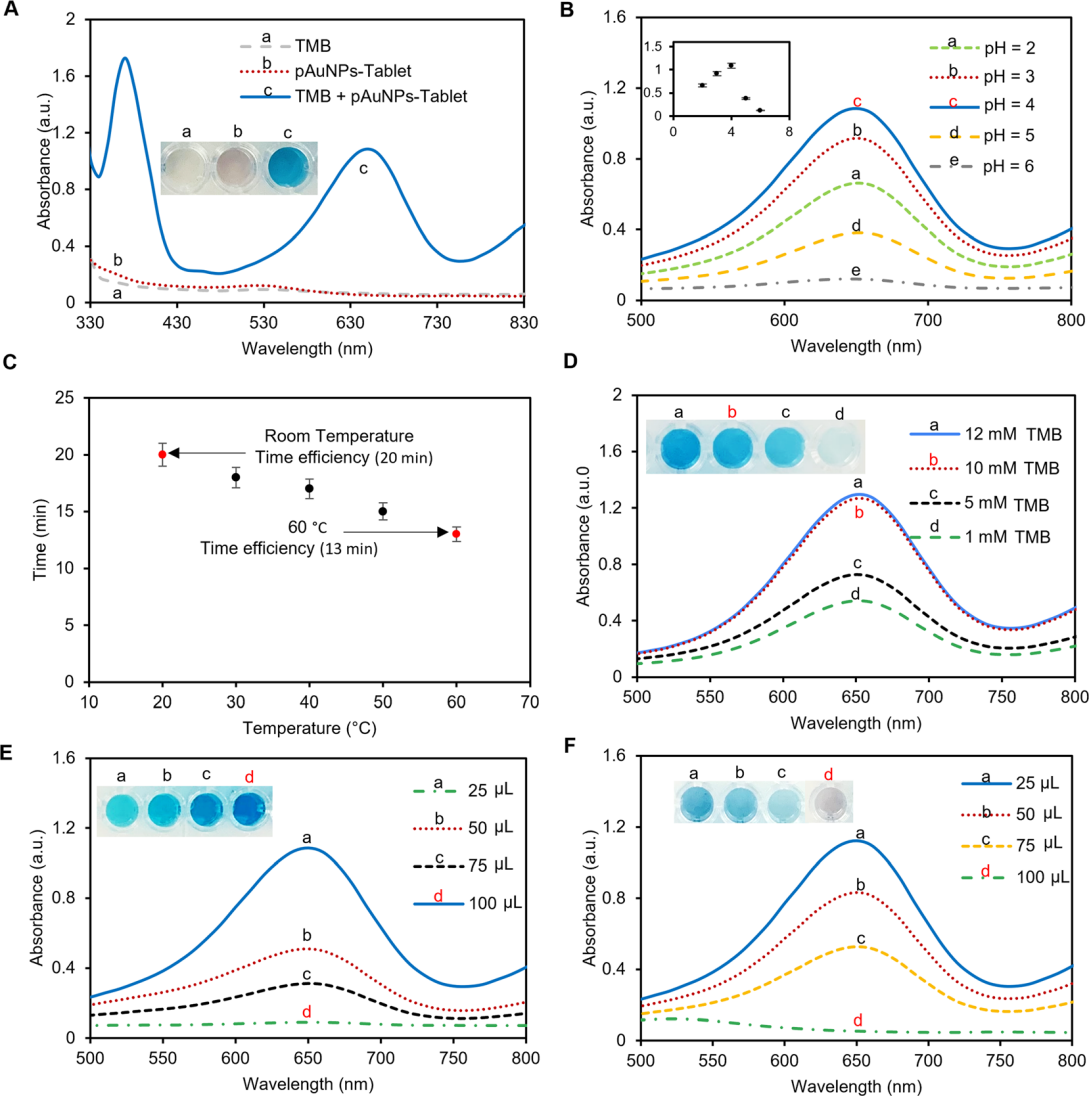
Optimization of the experimental condition for cysteamine detection. (A) UV-vis spectroscopy for TMB (colorless), pAuNP-Tablet (pinkish), and pAuNP-Tablet + TMB (blue), showing the peroxidase activity of the pAuNP-Tablet. (B) Effect of TMB's pH on the absorbance at 650 nm. Higher absorbances were observed at pH = 4 and 3. While a decrease in absorbance was noticed at pH = 2, 5, and 6. (C) Temperature effect on the increase in the reaction rate and the appearance of the blue color. It was noticed that the incubation of the samples at 40, 50, and 60 °C could accelerate the reaction and reduce the time to 17, 15, and 13 min, while more time was consumed at 20 and 30 °C. It was observed that a temperature >40 °C could break the inhibitory effect of cysteamine. Thus, the room temperature was chosen to perform the tests. (D) Optimizing the TMB concentration using 1, 5, 10, and 12 mM. The absorbance at 650 nm was less when using 1 and 5 mM, while an obvious increase was noticed using 10 mM. No further change in absorbance was observed using 12 mM. Hence, 10 mM TMB was used to perform the tests. (E) Effect of gold ratios (25, 50, 75, and 100 μL) to cast the tablets in the blue color formation. It was observed that 100 μL gave higher absorbance at 650 nm. Therefore, 100 μL was used to prepare the pAuNP-Tablet. (F) Inhibitory effect using various ratios, 25, 50, 75, and 100 μL, of 200 μM of cysteamine, showing the maximum inhibition using 100 μL cysteamine. Hence, this ratio was applied to the rest of the tests.

According to previously reported studies, amino acids act as antioxidants^4^ and for catalyst poisoning.^50^ Furthermore, recent studies have demonstrated that amino acids could interact with the nanozyme, where there is a competition between amino acids and the substrates to bind to the surface of the nanozyme.^51^ As such, we investigated the capability of cysteamine to inhibit the oxidation of the TMB–H_2_O_2_ system. According to the above-mentioned theory, cysteamine competes with the TMB substrate to bind to the surface of the pAuNP-Tablet causing the formation of a pAuNP-Tablet–cysteamine complex. This complex should inhibit the blue color development of the solution of pAuNP-Tablet and TMB, as shown in Scheme 1B. Furthermore, in the current case, the catalytic activity of the pAuNP-Tablet does not improve with time in cases where the cysteamine concentration is high enough to block the initial activity, 100 and 200 μM. This suggests that cysteamine may act as an inhibitor of the catalytic activity of the pAuNP-Tablet. Likewise, it was noticed that a blue color was observed in the absence of cysteamine exhibiting a high peak at 652 nm, whereas, in the presence of cysteamine, a flat peak for the colorless/grey color solution was detected as recorded by UV-vis spectroscopy, as displayed in Fig. 2A.

### 3.3 Optimization of the cysteamine assay using the pAuNP-Tablet as a peroxidase sensor

To provide the best condition with high peroxidase activity of the pAuNP-Tablet, several reaction parameters such as the pH, temperature, and the ratio of the pAuNP-Solution to cast the pAuNP-Tablet were studied. Since the inhibition of the peroxidase-like activity of the pAuNP-Tablet is dependent on the cysteamine amount in the system, the ratio of cysteamine to cause the inhibition was also optimized.

#### 3.3.1 Effect of pH

The TMB substrate contains two amino groups that are linked to the benzene ring and vulnerable to acidic conditions. Consequently, the changes in pH significantly affect the enzyme–substrate interaction. The study involved testing the substrate at various acidic pH levels ranging from 2 to 6, using a Mcilvaine buffer. TMB is colorless, while oxTMB is blue, which can easily be monitored by measuring the absorbance at 652 nm. The blue color developed due to the formation of a charge transfer complex, indicating the peroxidase activity of the pAuNP-Tablet, as presented in Fig. 2B. The highest absorbance was recorded at pH 4, and the system continued to function until pH 5, while the solution turned colorless/faded grey at pH 6. As a result, pH 4 was selected for the subsequent experiments.

#### 3.3.2 Effect of temperature

Enzymatic reactions are greatly influenced by temperature, as enzymes are highly sensitive to changes in heat and may lose their efficacy when exposed to higher temperatures. While natural enzymes tend to denature rapidly when the temperature exceeds 40 °C, artificial enzymes such as pAuNP-Tablets can endure higher temperatures. To analyze the impact of temperature on the oxTMB color over time, different temperature ranges (20–60 °C) were studied. It was noticed that oxTMB color formation was slower at room temperature, taking 20 minutes, while the reaction occurred more quickly (13 minutes) as the temperature was raised to 60 °C. This could be attributed to the faster dissolution of the pAuNP-Tablet, which releases the nanoparticles more quickly, increasing their energy capacity and decreasing the reaction time for the blue color development.^52^ However, at a temperature above 60 °C, no further color change of the solution was observed, as shown in Fig. 2C. Next, we evaluated the temperature influence on the inhibition capacity of cysteamine. It was observed that a temperature above 40 °C can break the inhibitory effect of cysteamine, which might be due to the increase in the catalytic activity at higher temperatures due to particle collisions.^53^ Therefore, the room temperature was chosen to perform the rest of the experiments.

#### 3.3.3 Effect of the TMB concentration

The concentration of the substrate is a critical factor in determining the frequency of effective collisions within the substrate–enzyme complex.^54^ By following Michaelis–Menten's equation, increasing the substrate concentration can enhance the probability of enzyme–substrate interaction. To determine the optimal quantity of the TMB substrate that can produce a strong blue color, four different concentrations of TMB (1, 5, 10, and 12 mM) were evaluated using a single tablet of pAuNPs. As depicted in Fig. 2D, the intensity of the color gradually increased as the TMB concentration increased. The highest absorbance peak was observed at 10 mM TMB, and further concentration increments did not significantly alter the peak intensity. Consequently, 10 mM TMB was employed in all the experiments. This color development is due to the degree of specificity of the pAuNP-Tablet towards TMB and might be attributed to the negative surface charge on the pAuNPs.^48^

#### 3.3.4 Effect of the pAuNP ratio to cast the tablet sensor

Various proportions of pAuNP-Tablet were utilized to examine the influence of the nanozyme concentration on the catalytic activity of the TMB–H_2_O_2_ system. Typically, a volume of 100 μL of pAuNP-Solution was dispensed to form a single tablet, but for optimization investigations, quantities of 25, 50, 75, and 100 μL of pAuNP-Solution were also employed to produce quarter, half, and three-quarter tablets. The findings revealed that the quantity of pAuNP-Tablet had a direct impact on the catalytic activity and the formation of the blue color. A smaller amount of AuNPs would partially catalyze the reaction, resulting in a light blue color, as depicted in Fig. 2E. However, the development of a blue color with 1 tablet indicated a desirable color change. Therefore, a 100 μL ratio, one tablet, was utilized to perform the experiments.

#### 3.3.5 Effect of cysteamine ratios

It is commonly understood that ˙OH is produced *via* the reaction of peroxidase-like mimics with H_2_O_2_, which can facilitate the oxidation of peroxidase substrates. According to the literature, competition between amino acids and the substrate for the surface of nanozyme is the main reason for inhibition.^55^ Based on the principles of enzyme kinetics, these inhibitors bind to the natural enzyme with a perceived affinity that is similar to the concentration of the enzyme's active sites. This suggests that when the concentration of the inhibitor increases, there will be a point where the enzyme activity is entirely lost. In the case of nanozymes, the active site is believed to be the surface atom, which may result in numerous active sites within a single nanozyme particle. Therefore, to completely deactivate a single nanozyme, a high concentration of inhibitor molecules would be necessary to drive the reaction. To further investigate the catalytic mechanism of the peroxidase-like activity of the pAuNP-Tablet, cysteamine was utilized as an inhibitor that formed a stable complex on the surface of the pAuNP-Tablet, which inhibits the oxidation of TMB. Based on the above-mentioned facts, the concentrations and ratios of cysteamine have a significant impact on the quantity of oxTMB in the system. As such, different ratios (25, 50, 75, and 100 μL) of 200 μM cysteamine were employed to assess the inhibition of the catalytic activity of the pAuNP-Tablet. It was observed that the maximum inhibition was achieved using 100 μL of cysteamine. Thus, this ratio was used for the detection of cysteamine, as shown in Fig. 2F.

### 3.4 Inhibition of the peroxidase-like activity of the pAuNP-Tablet in the presence of cysteamine

**Fig. 3:**
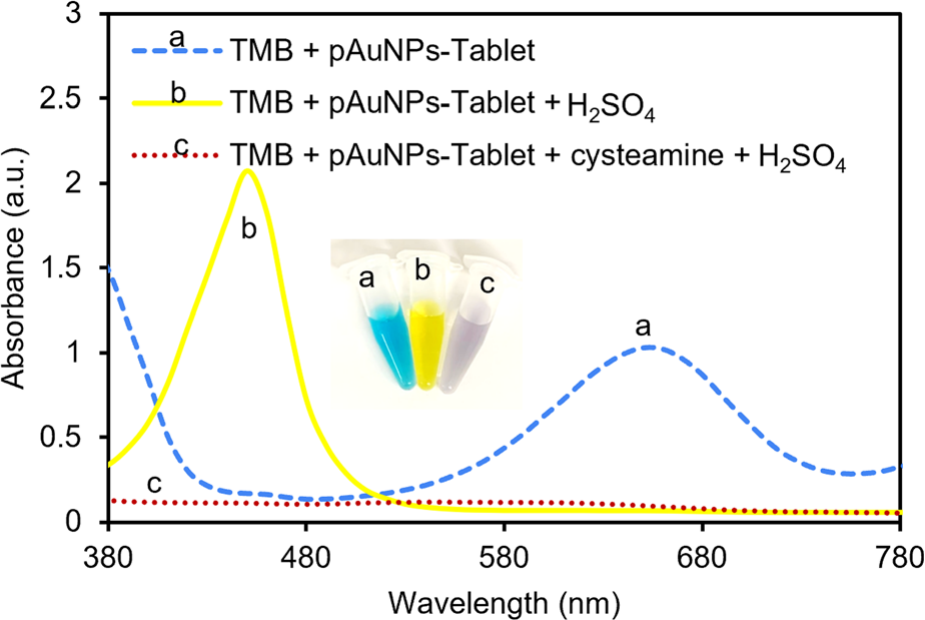
TMB response in the presence and absence of cysteamine and the effect of using sulfuric acid (H_2_SO_4_) as a color indication for oxTMB. In the absence of cysteamine and the presence of the pAuNP-Tablet, a high absorbance peak was observed at 652 nm. While no peak was observed when 200 μM of cysteamine was added to the system. The effect of using H_2_SO_4_ on TMB + pAuNP-Tablet and TMB + pAuNP-Tablet + cysteamine. The TMB color was shifted to 450 nm in the system containing TMB + pAuNP-Tablet, whereas adding H_2_SO_4_ to TMB + pAuNP-Tablet + cysteamine did not cause any color change since oxTMB was not present in the system.

The peroxidase activity of the pAuNP-Tablet was verified by introducing a 1 M H_2_SO_4_ stop solution, which quickly stopped the oxidation process of TMB and resulted in the formation of a yellow color. The test is based on the hypothesis that oxTMB (blue color) should be produced in the presence of the pAuNP-Tablet, whereas the presence of cysteamine prevents the formation of oxTMB. As a result, a change in the color of oxTMB from blue to yellow by H_2_SO_4_ can be used as an indicator of the presence of oxTMB. Since introducing cysteamine obstructed the active sites on the surface of the pAuNP-Tablet, oxTMB could not be formed. As a result of this inhibition, the absorbance peak of pAuNP-Tablet + TMB shifted from 652 to 450 nm, while no peak was observed for the TMB–pAuNP-Tablet system with cysteamine, as shown in Fig. 3. Thus, the yellow color intensity caused from the addition of H_2_SO_4_ is directly proportional to the amount of oxTMB, which was not produced when cysteamine is present.

### 3.5 Reaction kinetics in the presence and absence of cysteamine

**Fig. 4:**
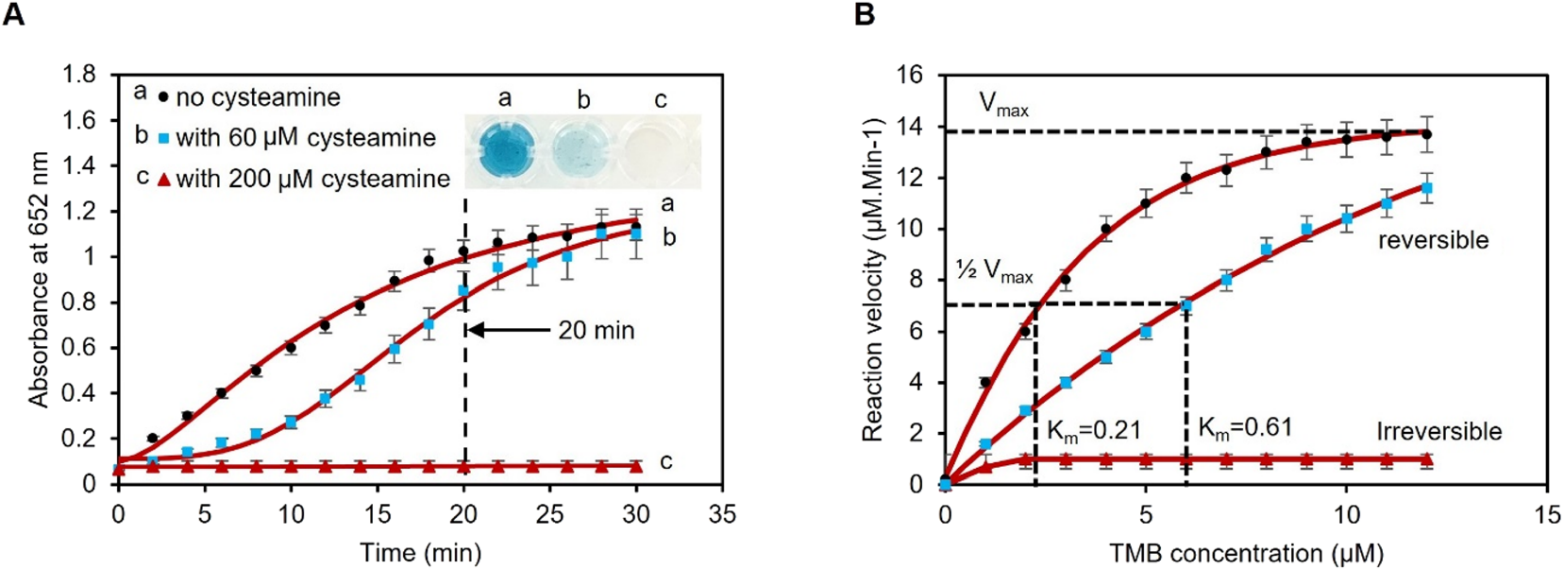
Reaction kinetics in the presence of 0, 60, and 200 μM of cysteamine. (A) Kinetics study shows a detection time of 20 min. The reaction solution in the absence of cysteamine converted turned completely blue (higher absorbance), while the solution containing 60 μM cysteamine shows less blue color intensity (less absorbance). However, no color transformation was achieved using 200 μM cysteamine, which confirms the absence of oxTMB. (B) Michaelis–Menten's parameters were obtained when using 0, 60, and 200 μM of cysteamine according to three replicas. A reversible (competitive) mechanism (blue squares) was observed when using 60 μM of cysteamine, while the inhibition was irreversible (non-competitive) using 200 μM of cysteamine (red triangles).

To set the assay time, the time for blue color saturation in the absence and presence of cysteamine was measured by UV-vis spectroscopy over a period of 30 minutes. The kinetics study for the oxidation of TMB using 1 mM H_2_O_2_, 1 pAuNP-Tablet, and 0, 60, and 200 μM of cysteamine was investigated. The results indicate a time-dependent kinetics analysis of the catalytic reaction between the pAuNP-Tablet and TMB substrates at both concentrations (0 and 60 μM) of cysteamine. Based on the findings, the activity using 0 μM cysteamine proceeds rapidly and reaches saturation at 20 minutes, as shown in Fig. 4A. While using 60 μM cysteamine, the reaction took a longer time of ∼2 hours till saturation. However, the reaction containing 200 μM of cysteamine showed no color development and remained permanently unchanged. This leads to further investigation of the type of inhibition (reversible or irreversible) to occur in the presence of 60 and 200 μM cysteamine. If we consider the potential influence of the effect of cysteamine concentration on the steady-state kinetics of Michaelis–Menten's parameters (*K*_m_ and *V*_max_) of the pAuNP-Tablet, we should assume to see no alteration in *K*_m_, as the irreversible binding of 200 μM cysteamine to the pAuNP-Tablet in the initial phase should not influence the instantaneous binding of substrate molecules to the surface of the pAuNPs. Conversely, considering that a reduced number of liberated surface atoms will be accessible to interact with the substrate, it is expected that *V*_max_ for substrate oxidation catalyzed by nanozyme will diminish.^56^ To confirm this theory, *K*_m_ and *V*_max_ were determined in the presence and absence of cysteamine, as illustrated in Fig. 4B. Based on this assumption, a decline in the *V*_max_ value of TMB substrates as the concentration of cysteamine is enhanced was noticed, while *K*_m_ remains unchanged when using 200 μM cysteamine, whereas different *K*_m_ and *V*_max_ were obtained using 60 μM cysteamine, indicating a reversible inhibition phenomenon. These results lead to the observation that double types of inhibition occurred, which is dependent on the concentration of cysteamine. Overall, in contrast to natural enzymes, where one inhibitor molecule typically leads to the deactivation of a single enzyme molecule by interacting at the active site, in the case of nanozymes, the surface atoms of the nanoparticles perform the same function as the enzyme's active sites.^28^ When the pAuNP-Tablet is exposed to low concentrations of cysteamine, it experiences only partial loss of activity as the surface of pAuNPs is not completely blocked. However, higher concentrations of cysteamine lead to a complete loss of catalytic activity. According to the enzyme kinetics theory, we observed that the affinity of the nanozyme to its substrate (*K*_m_) remained constant, while the catalytic efficiency decreased. This indicates that more than one cysteamine molecule binds to the surface of each pAuNP-Tablet, obstructing the surface atoms.

To further confirm the reversible/irreversible inhibition of the pAuNP-Tablet by 60 and 200 μM of cysteamine, we performed FTIR spectroscopy after centrifugation and washing of the colloidal pAuNP-Tablet–cysteamine complex. The results are presented in Fig. S3.† The FTIR spectrum of the washed pAuNPs exhibited extra peaks arising from the –SH bending and –NH stretching vibrations, which were absent in the case of the pristine pAuNP-Tablet. Moreover, the DLS analyses were performed to study the *ζ*-potential and hydrodynamic diameters of the same colloidal complex, as presented in Fig. S4.† The analyses show a decrease in *ζ*-potential to −2 mV and an increase in the hydrodynamic diameter of the nanoparticles to 785 nm with PDI% of 46% of the nanoparticles, which can be due to the formation of the pAuNP-Tablet–cysteamine complex. These findings confirm the potent affinity of cysteamine towards the pAuNPs, indicating the establishment of a durable complex between the nanozyme and inhibitor using a concentration of 200 μM. While no pAuNP–cysteamine complex was observed using a 60 μM concentration of cysteamine resulting in reversible inhibition. Next, we performed the catalytic activity test in the presence and absence of 0, 60, and 200 μM cysteamine. Briefly, the test is based on studying the conversion of 4-nitrophenol (4-NP) (yellow) to 4-aminophenol (4-AP) (colorless) in the presence of a catalyst. As displayed in Fig. S5A,† very fast conversion was achieved using 100 μL pAuNP-Solution (=1 pAuNP-Tablet), while slow conversion was observed using 25 μL pAuNP-Solution (=quarter tablet). The catalytic activity in the presence and absence of 200 μM cysteamine is shown in Fig. S5B.† The conversion of 4-NP into 4-AP was fully completed using the pAuNP-Tablet alone, while the conversion was blocked in the presence of 200 μM cysteamine, as shown in the reaction rate presented in Fig. S5C.† Moreover, the Langmuir–Hinshelwood model was used to calculate the apparent reaction constant (*k*_app_). The *k*_app_ value is higher in the case of 0 μM cysteamine, while a small value was observed using 200 μM cysteamine, suggesting that the pAuNP-Tablet–cysteamine complex prevented the conversion of 4-NP into 4-AP.

### 3.6 Colorimetric detection of cysteamine in buffer and artificial serum

**Fig. 5:**
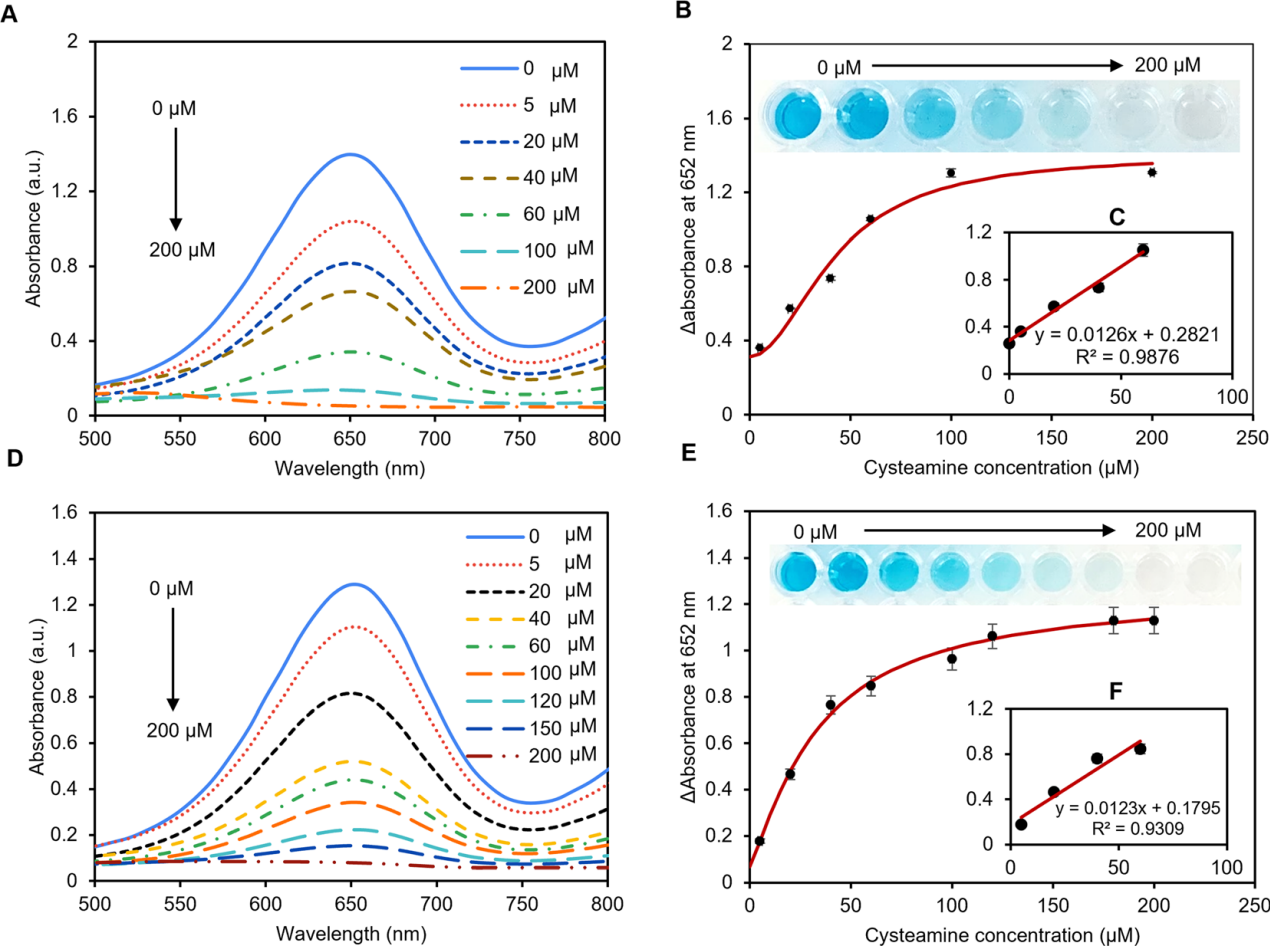
Detection of cysteamine in phosphate buffer and artificial serum. (A) UV-vis spectra of TMB in phosphate buffer pH = 7 in the presence of a different concentration of cysteamine (0–200 μM). (B) The calibration curve was obtained using various concentrations of cysteamine. (C) The linear range of the curve shows good linearity between (0–40 μM) of cysteamine. The LoD was calculated using the 3*σ*/slope method and was found to be 69 μM. (D) UV-vis spectra of TMB in artificial serum pH = 6.8 in the presence of a different concentration of cysteamine (0–200 μM). (E) The calibration curve was obtained using various concentrations of cysteamine. (F) The linear range of the curve shows good linearity between (0–40 μM) of cysteamine. The LoD was measured using the 3*σ*/slope method where the value was 80 μM. Δ*A* = (*A*_652 nm, absence_) − (*A*_652 nm, cysteamine_).

The colorimetric detection of cysteamine has been explored using the peroxidase-mimic activity of the pAuNP-Tablet. The reaction involves oxidation-mediated TMB and the pAuNP-Tablet in the presence of H_2_O_2_. TMB was used to capture the electron transfer from the catalyst, which was indicated by the development of the blue color of the system. This blue color of oxTMB is due to the establishment of a charge transfer complex that cannot form without a peroxidase catalyst. Hence, the pAuNP-Tablet was used as a peroxidase catalyst in the cysteamine assay. In initial experiments, cysteamine was detected in 0–200 μM concentrations in a buffer medium using 10 mM TMB. The blue color intensity of oxTMB was quantified by recording the absorption spectra at 652 nm. There is an obvious decrease in *λ*_max_ at 652 nm with the increase in cysteamine concentrations. While higher peaks appeared using (5–40 μM) cysteamine, which became less gradual with the increase in the concentration of cysteamine until a fully flat peak appeared with 200 μM, as shown in Fig. 5A. However, the absorbance *λ*_652_ remained unaffected with the further increase in cysteamine concentration, indicating the complete blockage of oxidation of the TMB substrate. A calibration curve of the enzyme mimic activity of the pAuNP-Tablet was plotted against cysteamine concentrations on the *x*-axis and a change in absorbance (Δ*A*) values at 652 nm on the *y*-axis resulting in a hyperbolic curve, which was fitted using the Hill model as shown in Fig. 5B. The relative absorption intensity is linearly proportional to the concentration of cysteamine at the first four concentrations, as displayed in the inset of Fig. 5C. with an LoD of 69 μM. Once the peroxidase behavior of pAuNP-Tablet was confirmed for cysteamine detection in PB, the same protocols were followed in artificial serum to acquaint with close-to-real-world situations. Next, the peroxidase-mimic activity of the pAuNP-Tablet was evaluated using different cysteamine concentrations (0–200 μM) in the artificial serum medium. The lower cysteamine concentrations <40 μM produced a high-blue color intensity solution, showing a plateau at 652 nm in the UV-vis spectrum. The reaction media turned to faded blue color progressively with the increase in cysteamine concentrations until 200 μM, as shown in Fig. 5D. The color of the TMB solution was changed according to the cysteamine concentration, which was used to calculate the amount of cysteamine in the calibration curve. The curve was plotted considering the relative change in absorbance intensity (Δ*A*) at 652 nm *versus* the cysteamine concentrations using the Hill model. An LoD of 80 μM was calculated in artificial serum, as shown in Fig. 5E. At lower concentrations, *λ*_652_ was linearly proportional to the concentration of cysteamine, as displayed in Fig. 5F.

In conclusion, our newly proposed pAuNP-Tablet sensor efficiently detected cysteamine in different media using the peroxidase catalytic property of AuNPs. Cysteamine that contains a thiol group is an essential amino acid that was found to be the only treatment for cystinosis.^57^ However, abnormal concentrations of cysteamine are implicated in a variety of health conditions. Therefore, it is highly important to report changes in cysteamine concentrations *via* real-time monitoring. Hence, the pAuNP-Tablet would be a potential platform in the disease diagnostic and detection field. Moreover, the pAuNP-Tablet sensor has the advantages of portability, low cost, user-friendliness, and colorimetric read-out, which make it a good candidate for point-of-care analytical devices.

### 3.7 Selectivity analysis towards cysteamine detection

**Fig. 6:**
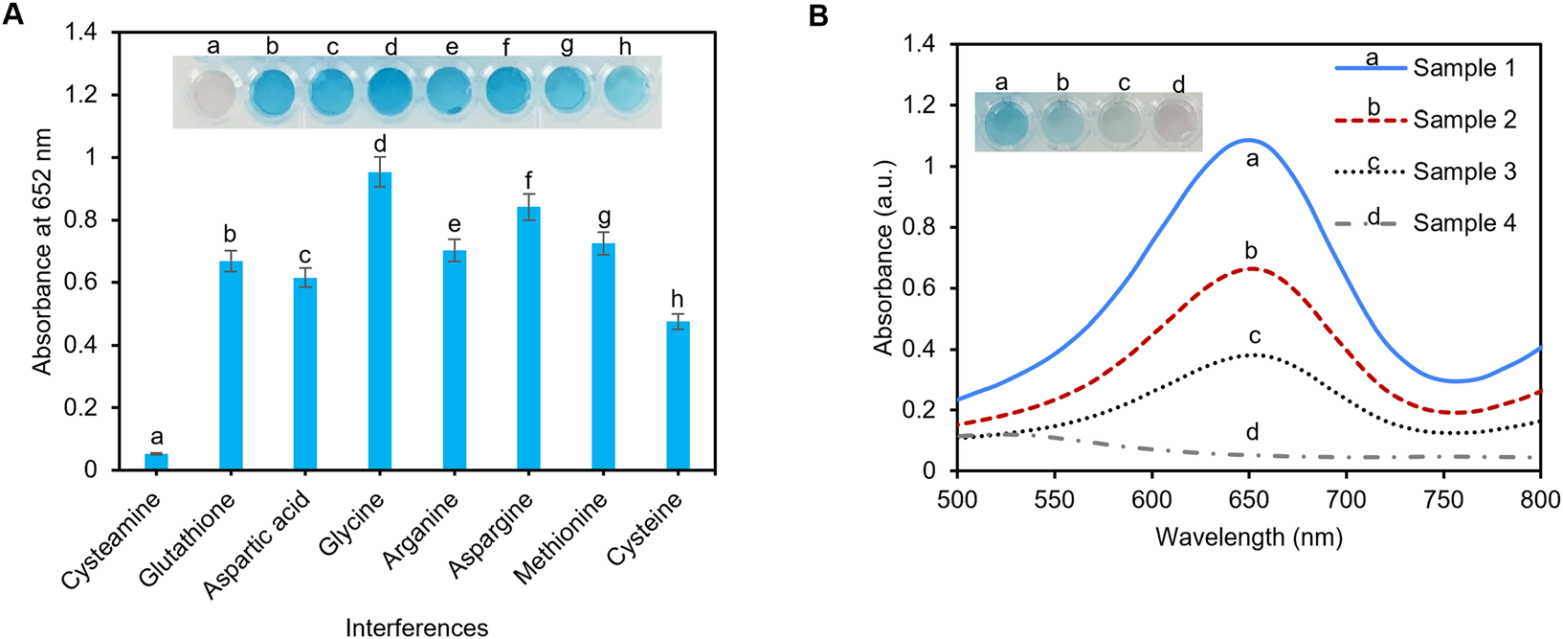
The interference studies and the real sample testing. (A) The selectivity of the sensor in the presence of other amino acids shows that other amino acids do not interfere with the sensor specificity for cysteamine. (B) UV-vis peaks at 652 nm of the samples before spiking indicating a higher peak for (sample 1: healthy individual) while a decrease in peaks was observed for samples 2, 3, and 4 which are samples taken from patients with cystinosis (*n* = 3).

To investigate the specificity of the pAuNP-Tablet sensor toward cysteamine detection, other amino acids including methionine, asparagine, glycine, glutathione, aspartic acid, cysteine, and arginine were tested in an artificial serum sample. For this study, 100 μM of each amino acid was incubated with the pAuNP-Tablet separately for 1 min followed by the addition of TMB and H_2_O_2_ solutions. There was an obvious change in the TMB color in the presence of other amino acids, while the TMB color remains colorless-grey in the presence of cysteamine, as illustrated in Fig. 6A. The absorbance spectra of the interferences are presented in Fig. S6.† Hence, these results confirm the specificity of our pAuNP-Tablet sensor for the detection of cysteamine.

### 3.8 Determination analysis of cysteamine in human serum

To evaluate the practical suitability of our suggested pAuNP-Tablet sensor, cysteamine detection was accomplished in human serum samples. Four samples (sample 1: healthy individual; samples 2, 3, and 4: cystinosis patients) were donated by a clinical lab in Montreal, Canada. For the detection of cysteamine, the time of sample collection is critical. Therefore, the samples were collected from patients after 30–60 min of digestion of 1200 mg of the Cystagon drug.^9^ It is worth mentioning that the serum samples were used without any treatment and stored in test tubes in well-closed plastic bags at −20 °C and used within three days. The samples were checked for cysteamine levels without spiking, as presented in Fig. 6B. The findings confirmed the presence of different concentrations of cysteamine. The obtained color development of oxTMB varied from less intense or faint blue in the case of samples 2, 3, and 4 to a very intense blue color as in sample 1. Finally, to assess the applicability of the pAuNP-Tablet for real-world applications, samples 1, 2, and 3 were spiked with cysteamine 100, 60, and 70 μM, respectively. As shown in Fig. S7† with the blue squares, red triangles, and green diamonds, the results of the measured intensities for the spiked samples followed the trend in the calibration curves, with the calculated recovery values of 105, 103.8, and 102% for sample 1, 101, 102, and 104% for sample 2, and 91, 94, and 98% for sample 3, respectively. Likewise, the real samples exhibited outstanding precision, as evidenced by the % RSD measurements of less than 2% for each of the three repeated data points. These results showed the applicability of the pAuNP-Tablet as a sensor for complex matrices in real-world applications.

### 3.9 Stability of the pAuNP-Tablet

The stability of the pAuNP-Tablet was studied for 16 months taking the absorbance spectra (3 readings) at 520/650 nm. The results are presented in Fig. S8.† Our tablets exhibited ultra-stability to date, which makes them an attractive platform over other point-of-care analytical devices.

## 4. Conclusion

The present research demonstrated the catalytic activity of a pAuNP-Tablet as a peroxidase mimic. The tablet nanozymes showed high specificity towards oxidation of a positively charged TMB substrate. The effects of other reaction parameters such as pH, temperature, TMB concentrations, pAuNPs, and cysteamine ratios were further investigated. Different media of phosphate buffer and artificial human serum were tested to quantify the amount of cysteamine. The kinetics analyses using the Michaelis–Menten model were also employed to study the inhibition types. Importantly, our study for the first time establishes cysteamine to exhibit two features as a reversible/irreversible inhibitor to temporarily/permanently block the catalytic activity of the pAuNP-Tablet nanozyme. Considering that surface atoms function as active positions in nanozymes, it was observed that the cysteamine concentrations had an impact on the suppression of catalytic activity. The specific nature of this interaction allowed the fabrication of a simple colorimetric sensor for cysteamine detection using the pAuNP-Tablet. As a proof of concept, sulfuric acid was used to confirm the inhibition by quantifying the amount of oxTMB through the formation of yellow color products. Our tablet detected low levels of cysteamine with LoD values of 69.04 and 82.9 μM in PB and artificial serum samples, respectively. Other potential interferences were used for the selectivity of the sensor towards cysteamine among other amino acids. The recovery percentages (% R) in real human samples show the method's validity with values ranging from 91 to 105% in real human serum samples. The pAuNP-Tablet showed ultra-stable properties for up to ∼16 months compared to the pAuNP-Solution, which enhances its applications as a point-of-care portable sensor. All in all, the combination of gold nanoparticles and pullulan in the tablet platforms provides numerous distinctive advantages including transportability, simple fabrication, low cost, high selectivity and sensitivity, and naked-eye visibility. Our study results provide strong evidence that the pAuNP-Tablet has a high potential for use in the routine clinical diagnosis of cysteamine overdoses in real human serum samples.

## Conflicts of interest

The authors declare no known competing financial interests.

## Supplementary Material

RA-013-D3RA03073C-s001
