# Supplementary material for: User-friendly and ultra-stable all-inclusive gold tablets for cysteamine detection
Source: RSC Adv. 2023 Jun 29;13(28):19638–50. doi: 10.1039/d3ra03073c (PMC10308203; doi:10.1039/d3ra03073c)
Supplement: RA-013-D3RA03073C-s001 [file RA-013-D3RA03073C-s001.pdf]

Electronic Supplementary Material (ESI) for RSC Advances.
This journal is © The Royal Society of Chemistry 2023

Supporting information

User-friendly and ultra-stable all-inclusive gold tablets for cysteamine detection

Muna Al-Kassawneh[1], Zubi Sadiq[1] and Sana Jahanshahi-Anbuhi[1,*]

[1]Department of Chemical and Materials Engineering, Gina Cody School of Engineering, Concordia University, Montréal, Québec, Canada

[*]Address correspondence to Sana Jahanshahi-Anbuhi, sana.anbuhi@concordia.ca

Content

Figure S 1. The AFM images of the pAuNPs-Tablet particles showing height trace of tablet surface........2
Figure S 2. The UV-vis spectra of pAuNPs-Tablet in the presence of (200 and 500 µM) cysteamine.........3
Figure S 3. The FTIR analyses after centrifugal and washing of the pAuNPs-Tablet-cysteamine solutions. 3
Figure S 4. The DLS analyses of the pAuNPs-Tablet-cysteamine complex...4
Figure S 5. The catalytic activity test using the conversion of 4-Nitrophenol (4-NP) (yellow) to 4-Aminophenol (4-AP) (colorless) in the presence of pAuNPs-Tablet.. 5
Figure S 6. The absorbance spectra of other amino acids that might be present in human serum. 6
Figure S 7. Analytical performance of the tablet-based sensor in real human serum samples....................6
Figure S 8. The stability analyses of pAuNPs-Tablet showing the ultra stable properties compared to pAuNPs-Solution over a period of ~16 months...7

List of abbreviations:

AuNPs	gold nanoparticles
pAuNPs-Solution	pullulan capped gold nanoparticles solution
pAuNPs-Tablet	pullulan capped gold nanoparticles tablets
%R	Recovery percentage
%RSD	Relative standard deviation
TMB	3,3', 5,5'-tetramethylbenzidine

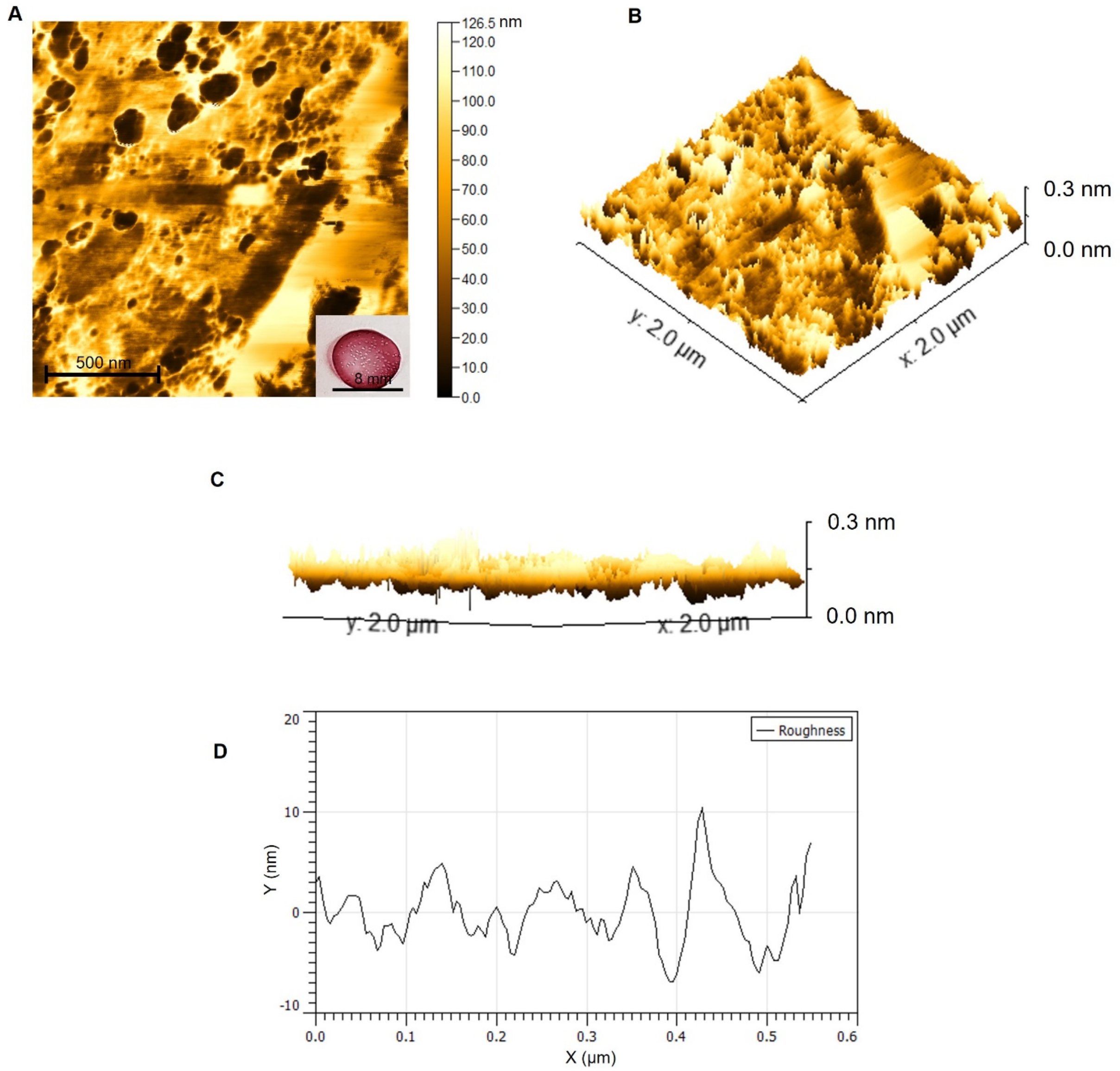

Figure S 1. The AFM images of the pAuNPs-Tablet particles show a height trace of the tablet surface. **A)** 2D image of height profile of pAuNPs-Tablet in an amplitude trace; **B and C)** 3D image of height profile of A; **D)** Height distribution as surface roughness and texture description along the black line area of image A. The average roughness was ~1.8 nm, the average maximum height of the roughness was ~10 nm, the average maximum roughness valley depth was ~6.6 nm, the maximum peak to valley roughness was ~18 nm and the waviness average was ~1.15 nm. The results confirmed that the strong binding between pullulan and AuNPs occurred.

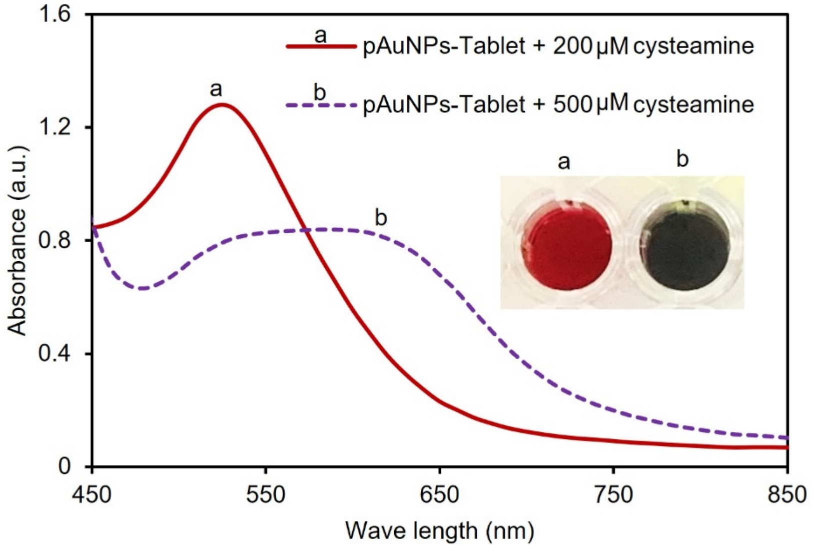

Figure S 2. The UV-vis spectra of pAuNPs-Tablet in the presence of (200 and 500 µM) cysteamine. A 200 µM of cysteamine could not cause the aggregation of pAuNPs-Tablet while 500 µM caused the aggregation. Considering the higher concentrations of cysteamine cause aggregation of pAuNPs-Tablet based on LSPR, the peroxidase-like activity method is the resolution for cysteamine detection to achieve lower LoD.

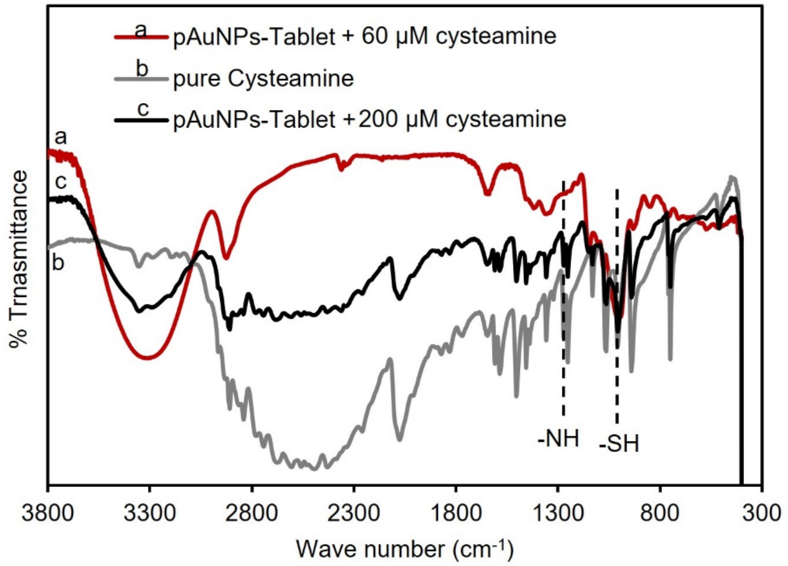

Figure S 3. The FTIR analyses after centrifugal and washing of the pAuNPs-Tablet-cysteamine solutions. The FTIR spectrum of the washed pAuNPs-Tablet + 200 µM cysteamine exhibited extra strong peaks arising from the -SH bend and -NH stretch due to the presence of pAuNPs-Tablet-cysteamine complex even after washing the catalyst. The absence of -SH and -NH peaks in the

pristine pAuNPs-Tablet + 60 μM cysteamine indicates the pAuNPs-Tablet-cysteamine complex formation is dependent on cysteamine concentration.

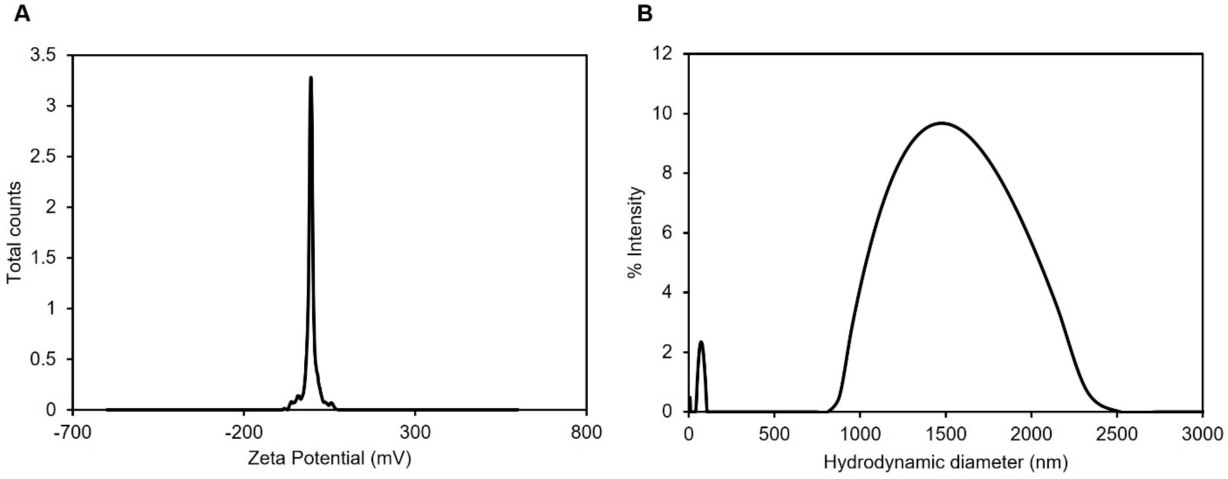

Figure S 4. The DLS analyses of the pAuNPs-Tablet-cysteamine complex. **A)** The ζ-potential analysis displays a decrease in ζ-potential value from-9 to -2 mV. **B)** An increase in the hydrodynamic diameter from 91.77 nm with PDI% of 24% to 785 nm with 46%, respectively indicating the formation of a complex of nanoparticles.

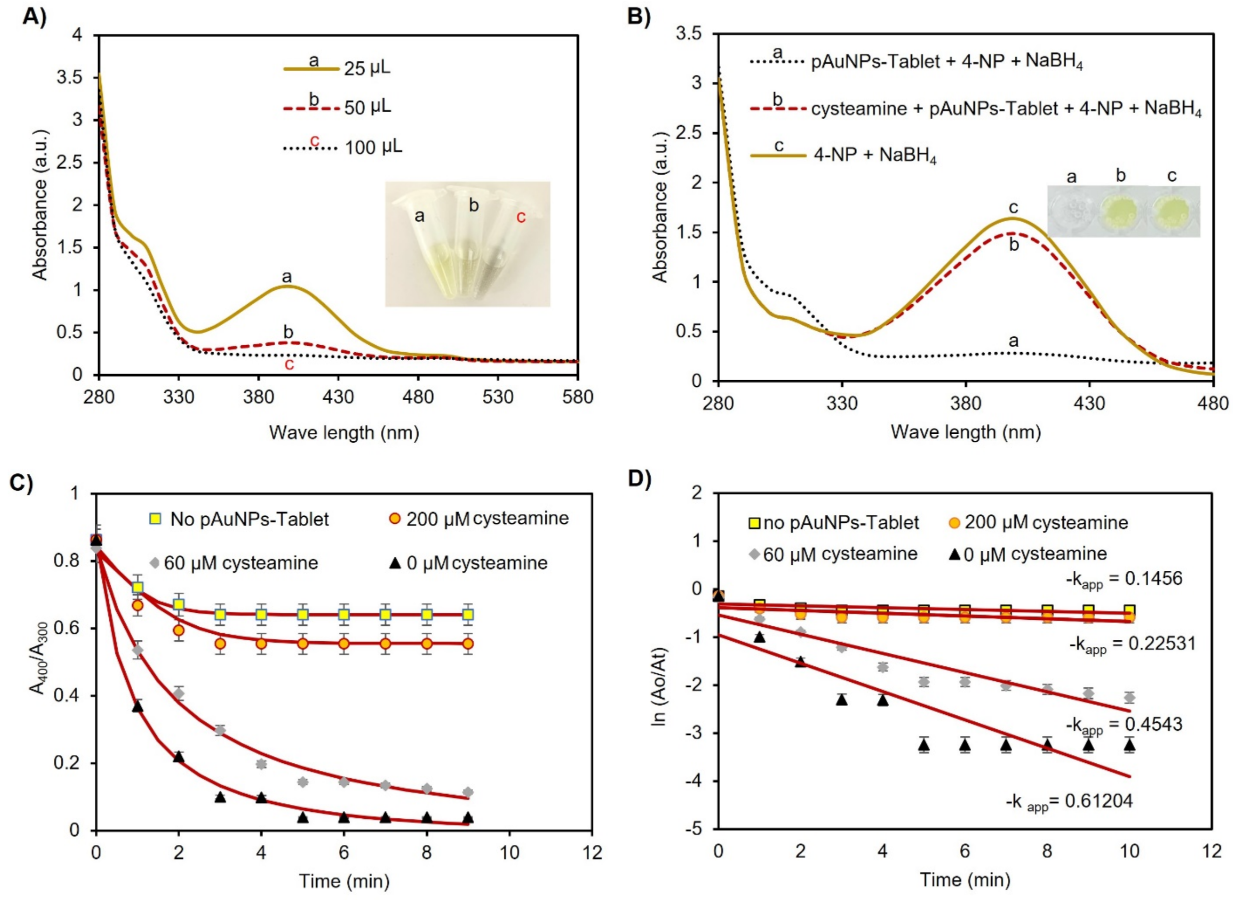

Figure S 5. The catalytic activity test using the conversion of 4-Nitrophenol (4-NP) (yellow) to 4-Aminophenol (4-AP) (colorless) in the presence of pAuNPs-Tablet. **A)** A higher catalytic activity was observed using 100 µM pAuNPs-Solution to cast pAuNPs-Tablet. **B)** The catalytic activity in the presence and absence of 200 µM cysteamine. The conversion of 4-NP into 4-AP was completed using pAuNPs-Tablet. While the conversion was blocked in the presence of 200 µM cysteamine. **C)** The reaction rate in the presence of (0, 60, and 200 µM) cysteamine shows a very fast conversion when 0 µM cysteamine. Whereas a slower reaction was noticed in the presence of 60 µM cysteamine. Almost no conversion was achieved in the case of 200 µM cysteamine which was comparable to the color of the blank. **D)** The Langmuir-Hinshelwood model to calculate the apparent reaction constant (k_{app}) shows a fast reaction in the case of 0 µM cysteamine while a very low (k_{app}) was observed in 200 µM cysteamine indicating that pAuNPs-Tablet-cysteamine complex prevented the conversion of 4-NP to 4-AP.

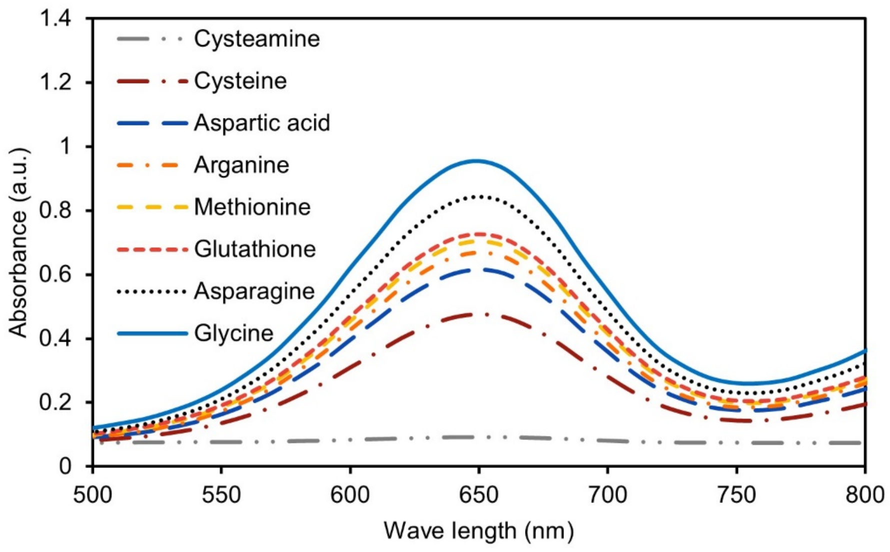

Figure S 6. The absorbance spectra of other amino acids that might be present in human serum. The results display that only cysteamine could inhibit the peroxidase-like activity of pAuNPs-Tablet due to the high presence of cysteamine in human serum after 30 min of digestion of the cysteamine.

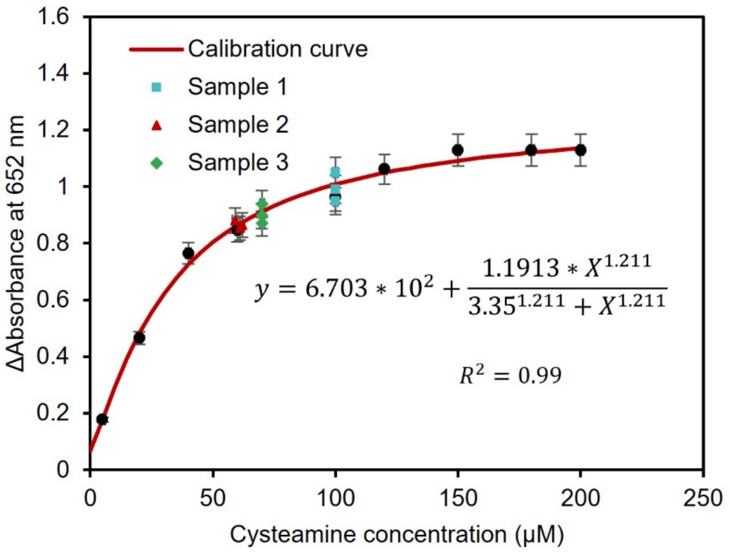

Figure S 7. Analytical performance of the tablet-based sensor in real human serum samples. The calibration curve was attained by testing the sensor with the known concentrations of 60, 70, and 100 μM cysteamine in real human serum samples. A Hill function was fit to the whole data with an $R^2 > 0.99$, showing the compatibility of the data with a standard saturation model. Each data point is the mean ± standard deviation of the replications (n = 3)

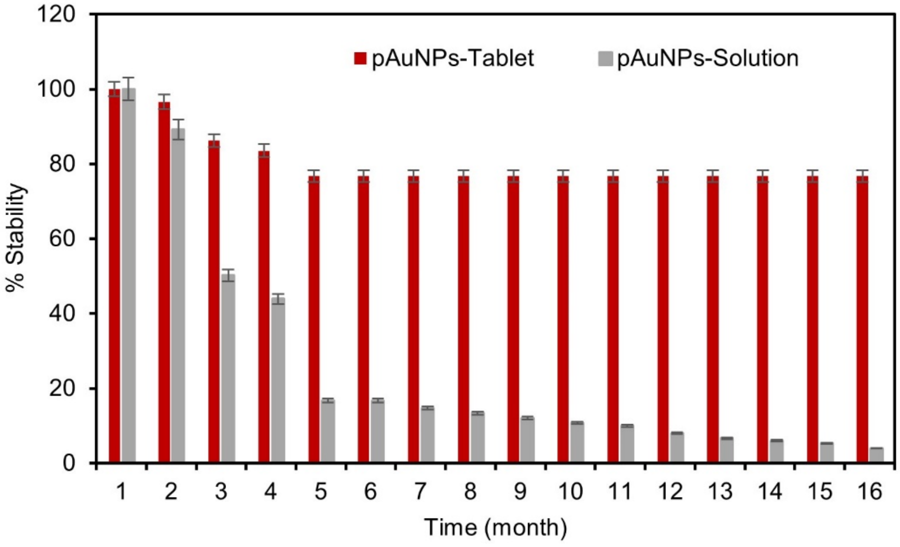

Figure S 8. The stability analyses of pAuNPs-Tablet show ultra-stable properties compared to pAuNPs-Solution over a period of ~16 months.
